# Aortic stiffness as a predictor of high-sensitivity cardiac troponin T levels at a chronic stage after ST-segment elevation myocardial infarction

**DOI:** 10.1186/1532-429X-17-S1-P127

**Published:** 2015-02-03

**Authors:** Hans-Josef Feistritzer, Gert Klug, Sebastian J Reinstadler, Benjamin Seidner, Johannes M Mair, Michael Schocke, Wolfgang-Michael Franz, Bernhard Metzler

**Affiliations:** University Clinic of Internal Medicine III, Cardiology and Angiology, Medical University of Innsbruck, Innsbruck, Austria; Departement of Radiology I, Medical University of Innsbruck, Innsbruck, Austria

## Background

Aortic stiffness is associated with early pulse wave reflection resulting in an increase of cardiac afterload and impairment of coronary perfusion. Experimental data show that high left ventricular pressure due to increased aortic stiffness is associated with enhanced myocardial cell death. We investigated whether aortic stiffness is related to high-sensitivity cardiac troponin T (hs-TnT) concentrations at a chronic stage 1 year after ST-segment elevation myocardial infarction (STEMI).

## Methods

Seventy-four patients underwent cardiac magnetic resonance imaging for the assessment of left ventricular function, morphology, infarct size and aortic PWV 12 months after acute STEMI. Blood samples were drawn at 12 months by peripheral venipuncture. Hs-TnT levels were measured by a commercially available immunoassay (Roche Diagnostics^®^).

## Results

hs-TnT concentrations (6.4 ng/L, IQR 5.0 - 8.6) were significantly associated with age (r = 0.417, p < 0.001), plasma creatinine levels (r = 0.257, p = 0.027), high-sensitivity-C-reactive protein levels (r = 0.281, p = 0.015) and aortic PWV (r = 0.435, p < 0.001). Multiple linear regression analysis revealed aortic PWV (ß = 0.349, p = 0.014) beside, plasma creatinine concentrations (ß = 0.288, p = 0.006) and diastolic blood pressure (ß = 0.243, p = 0.015) to be independently associated with hs-TnT concentrations (model: R = 0.622, p < 0.001).

## Conclusions

Aortic stiffness is an indicator of prognosis after myocardial infarction. The present study suggests an impact of aortic stiffness on hs-TnT concentrations at 1 year after STEMI.

## Funding

Austrian Society of Cardiology.

